# Development of the EPO-Score – a multivariable tool to predict adverse outcome in infants with perinatal asphyxia undergoing therapeutic hypothermia – a retrospective study

**DOI:** 10.3389/fped.2025.1627300

**Published:** 2025-08-06

**Authors:** Adriana van der Donk, Maria Schleier, Alisa Bär, Linda Mulzer, Regina Trollmann, Stephanie Schuessler, Steven Hébert, Gregor Hanslik, Joachim Woelfle, Heiko Reutter, Patrick Morhart

**Affiliations:** ^1^ Division of Neonatology and Pediatric Intensive Care, Department of Pediatrics and Adolescent Medicine, University Hospital Erlangen, Erlangen, Germany; ^2^ Department of Pediatrics, Pediatric Neurology, University Hospital Erlangen, Erlangen, Germany

**Keywords:** asphyxia, hypothermia treatment, hypoxic ischemic encephalopathy, neonatal outcome, cerebral palsy, Griffiths mental development scales

## Abstract

**Aim:**

Early outcome prediction in neonates with perinatal asphyxia receiving therapeutic hypothermia (TH) remains difficult. Although several studies have explored prognostic markers and proposed scoring systems, none of these tools has been adopted for routine bedside use to date. The present retrospective study aimed to design an early prognostic outcome score (EPO-Score). The score serves to identify patients at discharge, predicting severe adverse outcomes according to the Griffiths Mental Development Scales (GMDS) with one year.

**Methods:**

Perinatal data was collected from 44 infants with perinatal asphyxia who had received therapeutic hypothermia between 2010 and 2020 at the University Hospital Erlangen, Germany. 27 predictive variables were analyzed regarding their prognostic significance. Analysis showed significant correlations between 15 variables and their outcome. Outcome at one year was classified as favorable (GMDS DQ > 78) or severe adverse (DQ < 78, cerebral palsy, or death). We combined related variables into four indices: systemic injury, neurologic, liver and renal damage. A forward-looking step-by-step analysis revealed a model, explaining 62.1% of the variance in the outcome (R^2^ = 0.621; *p* < 0.001). Based on these results, we developed the EPO-Score and correlated the score to the follow-up assessment at one year.

**Results:**

A total of 32 (out of 44) infants met the inclusion criteria. 25 infants experienced a favorable outcome, 7 infants a severe adverse outcome. The EPO-Score integrates eight routine predictors. Average EPO-Score among all infants was 11 points (range 0–24). The EPO-Score showed significant association with the developmental outcome at one year of age (R^2^ = 0.421, *p* < 0.001). ROC-analysis demonstrated the EPO-Score's ability to distinguish between favorable and severe adverse developmental outcome, with a cut-off value of 13.5 (AUC = 0.926; 95% CI 0.831–1.00). Infants with a score of 14 or higher were classified as high-risk.

**Conclusion:**

EPO-Score underlines the correlation between the severity of early multi-organ involvement and severe adverse outcome, demonstrating a high predictive value within our study population. Early identification of patients with severe adverse outcome is important for optimizing neurodevelopmental therapies and providing family support. Nevertheless, external validation is required before the score can be implemented in routine clinical care.

## 1 Introduction

Despite numerous developments in neonatal care in recent years, perinatal asphyxia remains one of the leading causes for death or disability in term newborns worldwide (1). Due to an impaired placental or neonatal pulmonary gas exchange, vital organs such as the brain suffer from hypoxia and hypercapnia and/or reduced blood perfusion, with severe impact on physical and/or neurological outcome (2). With an incidence of approximately 3–5 per 1.000 term infants in technically developed countries, perinatal asphyxia represents a major risk factor for the development of hypoxic-ischemic encephalopathy (HIE) and severe neurologic damage. Moderate to severe HIE occurs in 0.5–1 live births (3, 4). It is therefore essential to identify and categorize high risk infants. At present, whole-body hypothermia is the only effective treatment strategy for infants with HIE (5) and is therefore included as standard treatment for HIE, when criteria are fulfilled (6, 7). The overall situation is extremely difficult for parents, which is reinforced by several uncertainties. Assessing the extent of injury and evaluating long-term prognosis in infants with HIE treated with therapeutic hypothermia (TH) remains challenging.

In previous studies, several predictive markers have been identified regarding the possible neurological outcome in infants with perinatal asphyxia associated with HIE (6, 8). However, most of these studies focused on single parameters. Only a few studies looked into combining parameters or tried creating a scoring system to categorize newborns, to some extend providing a glimpse into the future (9–11). Furthermore, many of these studies involve complex scoring systems which are difficult to integrate into clinical practice. The aim of the present study was to identify a suitable set of organs, clinical and laboratory markers, which together provide sufficient power to predict the neurological outcome, with the ability to differentiate between a favorable and a severe impaired neurological outcome. Against this background, we aimed to develop the Early Prognostic Outcome (EPO) Score - a multivariable model based on routinely collected clinical and laboratory data. The goal of the score is to help identify high-risk infants at the time of discharge from the Neonatal Intensive Care Unit (NICU), in order to support parent counselling, plan follow-up care, and consider early therapeutic interventions where needed.

## 2 Materials & methods

### 2.1 Study design

This is a monocentric retrospective study approved by the Ethics Committee of Friedrich-Alexander-University, Erlangen-Nürnberg (application number 22-345-B). All routinely collected data were retrieved from the hospital's electronic health-record and laboratory systems, where they had been entered prospectively for clinical care rather than research; no additional information was gathered specifically for this study.

### 2.2 Newborns/patients

**Figure 1:**
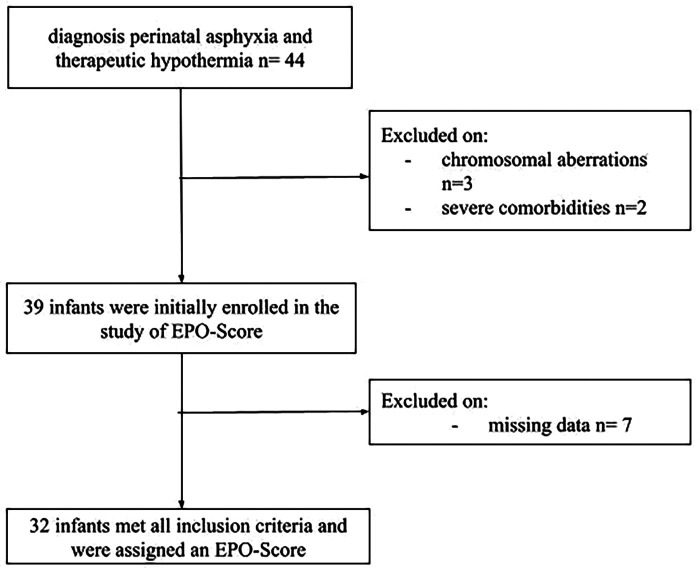
Flowchart of patient selection and final study cohort.

All infants with perinatal asphyxia associated with HIE receiving therapeutic hypothermia between 2010 and 2020 at the Neonatal Intensive Care Unit of the University Hospital in Erlangen, Germany were included. Perinatal asphyxia was defined as a condition of fetal distress (pathological CTG or fetal bradycardia or fetal blood gas analysis with arterial lactate >4.7 mmol/L or green amniotic fluid), and pH ≤7.0, or Base Excess ≥−16 mmol/L, or 5-minute APGAR-Score <6. Perinatal hypoxic-ischemic encephalopathy was assessed using the modified classification of Sarnat and Sarnat (12–14). Infants treated with TH met the following criteria: diagnosis of perinatal asphyxia, start of TH within 6 h of birth, gestational age ≥36 weeks, neurological abnormalities such as pathological amplitude-integrated electroencephalography (aEEG) or clinical signs of moderate or severe HIE in at least three of the six clinical categories according to Sarnat and Sarnat (12, 14) (Supplementary File S1) The initiation and administration of therapeutic hypothermia followed the national guidelines of the German Society of Neonatology and Pediatric Intensive Care (13). For the purpose of EPO-Score development, additional inclusion criteria specific to this study were applied: full documentation of blood values, MRI performed within the first 10 days of life and available neurodevelopmental data at one year of age. Newborns with significant comorbidities, such as severe malformations or chromosome aberrations and/ or incomplete data including perinatal clinical data, laboratory data and neurodevelopmental data at one year of age, were excluded from this study retrospectively (Figure 1).

### 2.3 Treatment, clinical examinations and follow-up

Hypothermia was performed using a cooling mat (Hirtz HICO Variotherm 550) and was conducted in accordance with current clinic guidelines (3, 13, 14). All infants had the following blood values obtained every 6 h within the first 24 h of cooling: lactate, pH, Base excess (BE), Creatine kinase (CK), Lactate dehydrogenase (LDH), Aspartate aminotransferase (AST), Alanine aminotransferase (ALT), serum creatinine, urea and Quick. Between 24 and 48 h postpartum and 48 h–72 h postpartum, blood values were obtained every 8 h. Furthermore, urine output was measured on the first five days. During the ongoing hypothermia treatment, blood gas analysis and clinical examinations were carried out at 0 h, 3 h, 6 h, 12 h after birth and then at least every 8 h. Besides aEEG monitoring, cranial ultrasounds were performed every 24 h, including Doppler sonography of the anterior cerebral artery. In addition, echocardiography was performed within the first 24 h. Further echocardiography studies were performed as needed. Further measurements after therapeutic hypothermia treatment included cranial magnetic resonance imaging (MRI) on day 5–7 after completed cooling (Supplementary Table S2). MRI brain scans were reviewed by a neuroradiologist who was blinded to early clinical data and assessed for the presence of injury patterns such as hemorrhage, edema or necrosis, or classified as showing no detectable injury. Last neuropediatric examination was carried out before discharge. After discharge, infants were linked to the outpatient clinic of the Division of Pediatric Neurology at the University Hospital Erlangen and were regularly examined regarding their motor and neurological development. For the present study, we considered the developmental outcome at 12 months of age using the German version of Griffiths Mental Development Scales (GMDS II) (15).

### 2.4 Outcome

Outcome was retrospectively assigned to patients based on their follow-up examination results using the German version of GMDS (15), presence of cerebral palsy and mortality. GMDS-II was administered in the outpatient clinic of the Division of Pediatric Neurology at the University Hospital Erlangen by a certified in-house occupational therapist who was blinded to the EPO-Score and early clinical data. Cerebral palsy diagnoses were confirmed at a separate visit by a pediatric neurologist, also blinded to predictor data. Mortality status was obtained from electronic medical records. Infants were retrospectively assigned to two categories - infants with a favorable outcome and severely affected infants, based on their assessment. Most severe outcome was defined by death. Infants who achieved a Developmental Quotient (DQ) > 78 across all subscales (foundations of learning, language and communication, eye and hand coordination, personal-social-emotional and gross motor) were classified as having age-appropriate development and were categorized into the favorable outcome group. Infants with a DQ < 78 or who developed cerebral palsy or who died because of perinatal asphyxia were assigned to the severe adverse outcome group.

### 2.5 Statistical analysis

Statistical analysis was performed with IBM SPSS Statistics, Version 29.0.1.0. Continuous, normally distributed data are presented as means with standard deviations, whereas medians and interquartile ranges are reported for continuous, non-normally distributed data. Spearman correlation coefficients were calculated to investigate the strength and direction of relationship between non-categorical variables. For categorical variables, a Chi-Square test was conducted to explore potential associations.

Correlation analysis was used to provide an overview of the associations between the different variables. To assess potential influencing factors, regression analysis was performed. Due to the small sample size and the presence of multicollinearity, various parameters were combined into indices that exhibited high correlation and could also be meaningfully combined in clinical practice. Since the variables were measured in different units, they were standardized using z-transformation. Overall, four indices were identified: (i) systemic injury, (ii) neurologic damage, (iii) liver damage, (iv) renal damage.

Multiple linear regression analysis was used to determine the direction and strength of influence of the created indices on neurodevelopmental outcomes and mortality. This analysis contributed to the development of the EPO-Score. All *p*-values <0.05 were considered statistically significant.

## 3 Results

### 3.1 Study selection and descriptive analysis

In this study a total of 44 infants were assessed of whom only 32 infants met all inclusion criteria. Three infants were excluded due to chromosomal aberrations (2 infants had trisomy 21, 1 infant had trisomy 13) and two infants were excluded due to severe comorbidities. In the end, seven infants were excluded because of missing follow-up data (Figure 1). All 32 infants received therapeutic hypothermia for 72 h without redirection of care. Among the 12 infants, excluded from analysis for not meeting the inclusion criteria, therapeutic hypothermia was discontinued in two cases, because of disseminated intravascular coagulation (DIC). In total, 27 routinely measured variables were examined. However, prior to analysis five candidate predictors [Troponin I, Creatine Kinase-MB (CK-MB), Brain Natriuretic Peptide (BNP), S100 protein, Ferritin] were excluded because each had more than 25% missing data, leaving 22 variable for further screening (Supplementary Table S3). An overview of the demographic and clinical neonatal characteristics can be found in Table 1. Griffiths Mental Development Assessment was available in 25 infants at a median age of 12 months (10–15 months). Median DQ was 95 (80–110). Three patients were diagnosed with cerebral palsy, two classified as level IV according to the Gross Motor Function Classification System (GMFCS) and one as level V. Four infants died due to poor medical condition following perinatal asphyxia. All infants assessed with the GMDS achieved a DQ > 78 and were therefore considered to have age-appropriate development. These infants were assigned to the favorable outcome group. In contrast, the three infants diagnosed with cerebral palsy and the four infants who died due to poor medical condition following perinatal asphyxia were classified as having a severe adverse outcome (Table 2). Overall, 7 of 32 infants (21.8%) were assigned to the severe adverse-outcome group. This figure refers to the entire cohort, which included all three Sarnat stages of hypoxic–ischaemic encephalopathy. When the data are stratified by grade, the adverse-outcome rates are 0% (0/4) for mild HIE I, 11% (2/18) for moderate HIE II, and 50% (5/10) for severe HIE III.

**Table 1:** Demographic and clinical neonatal characteristics.

Patient characteristics	All infants (*n* = 32)
Gestational age, weeks^a^	39 (36, 41)
Birth weight, grams^a^	3,205 (2,200, 4,690)
Mode of delivery	
Vaginal	7
Emergency cesarean	19
Vacuum extraction	6
Stage of HIE (classified according to Sarnat and Sarnat)	
1	4
2	18
3	10
Perinatal vital parameters	
pH^a^^,^^b^	6.86 (6.5, 7.38)
BE mmol/L^a^^,^^b^	−20.05 (−45.2, −3.8)
1 min APGAR^a^	1 (0, 6) *
5 min APGAR^a^	3 (0, 7) *
10 min APGAR^a^	5 (0, 8) *
Serial laboratory / organ-injury markers	
First arterial lactate mmol/L^a^	8.6 (1.3, 25)
Lactate 6 h mmol/L^a^	5 (1.1, 16)
Lactate 12 h mmol/L^a^	3.95 (0.9, 14.7)
Lactate 18 h mmol/^a^	4.0 (0.7, 20)
Lactate 24 h mmol/L^a^	3.15 (0.9, 21)
Lactate 30 h mmol/L^a^	2.75 (0.8, 16)
Lactate 36 h mmol/L^a^	2.5 (0.9, 13.9)
Peak CK U/L^a^^,^^c^	1,322 (198, 6,720)
Peak LDH U/L^a^^,^^c^	1,254 (432, 10,911)
Peak AST U/L^a^^,^^c^	182 (53, 6,414)
Peak ALT U/L^a^^,^^c^	56 (12, 3,597)
Peak serum creatinine mg/dl^a^^,^^c^	1.005 (0.66, 2.06)
Peak urea mg/dl^a^^,^^c^	32 (15, 59)
Quick^d^ at admission	38 (13, 71)
Clinical course / brain imaging	
Urine output (ml/kg/h)	
0–24 h of life^a^	2.45 (0.01, 8.92)
24–48 h of life^a^	3.97 (0, 10.07)
48–72 h of life^a^	4.88 (0.18, 9.31)
Duration of mechanical ventilation in h^a^	165.5 (117, 429)
Duration of catecholamines needed in h^a^	132 (0, 240)
MRI	
No brain damage	19
Brain damage	13

^a^ Data expressed as a median (IQR).

^b^ Data collected within first 3 h of life.

^c^ Data collected within first 48 h of life.

^d^ Data expressed as %.

*For all variables except APGAR 1/5/10 min, data was complete (*n* = 32), for APGAR 1/5/10 data was available of *n* = 27/26/25.

**Table 2:** Distribution of 12-month outcomes.

12-month outcomes	Favorable outcome	Severe adverse outcome
*n* = 32	25	7^a^

^a^ We had to conduct 4 deaths and 3 patients with cerebral palsy within the group “severe adverse outcome”.

To explore possible correlations between variables and outcome we calculated Spearman correlation coefficients and Chi-Square-tests. In this explorative correlation analysis, it was found that 15 out of 22 parameters showed a significant correlation with the outcome (Table 3) and were therefore examined in more detail. Moreover, analysis showed that some variables showed a high correlation, leading to high multicollinearity.

**Table 3:** Correlations between different parameters and outcome.

Parameter	Correlation coefficient	*p*-value
First arterial lactate	*ρ* = −0.451	0.010
Lactate at 6 h after birth	ρ = −0.479	0.006
Lactate at 12 h after birth	ρ = −0.463	0.008
Lactate at 18 h after birth	ρ = −0.475	0.006
Lactate at 24 h after birth	ρ = −0.487	0.005
Lactate at 30 h after birth	ρ = −0.536	0.002
Lactate at 36 h after birth	ρ = −0.520	0.002
Base Excess (first 3 h)	ρ = −0.651	<0.001
Peak LDH (first 48 h)	ρ = −0.364	0.040
Peak AST (first 48 h)	ρ = −0.377	0.034
Peak serum creatinine (first 48 h)	ρ = −0.487	0.005
Urine output (24–48 h)	ρ = −0.479	0.006
Duration of mechanical ventilation	ρ = −0.455	0.009
Stage of HIE (Sarnat and Sarnat)	χ^2^ (2) = 12.430	0.002
Brain damage on MRI	χ^2^ (2) = 13.095	<0.001

Highly correlated parameters were therefore combined into indices. The highest creatinine level within the first 48 h after birth and urine output between 24 and 48 h of life showed a significant correlation (*ρ* = −0.477, *p* = 0.006) and was therefore combined forming the **renal damage** index. Similarly, the first measured arterial lactate and the highest LDH value within the first 48 h after birth demonstrated a significant correlation (*ρ* = 0.422, *p* = 0.016) forming the index: **systemic injury**. Peak ALT and AST values within the first 48 h after birth strongly correlated (*ρ* = 0.798, *p* < 0.001), and were used to define the grade of **liver damage**. The fourth index, the **neurologic damage**, was derived from the significant correlation between MRI findings and the degree of hypoxic-ischemic encephalopathy [χ^2^(2) = 6.71, *p* = 0.035]. This approach reduces multicollinearity and enhances model stability by consolidating related variables into single indices. An overview of the medians and ranges of the four indices for each outcome group is presented in Table 4.

**Table 4:** Distribution of clinical indices by favorable vs. severe adverse outcome.

Indices	Parameters	Favorable outcome *n* = 25	Severe adverse outcome *n* = 7
Systemic injury	First arterial lactate	7.8 mmol/L (1.3–22)	19 mmol/L (8.6–25)
	Peak LDH (first 48 h)	1,094 U/L (432–3,147)	1,616 U/L (732–10,911)
Neurological damage	Stage of HIE	2 (1–3)	3 (2–3)
	Brain damage^a^ (MRI)	0 (0–1)	1
Liver damage	Peak AST (first 48 h)	161 U/L (53–897)	303 U/L (111–6,414)
	Peak ALT (first 48 h)	52 U/L (12–696)	70 U/L (17–3,597)
Renal damage	Peak serum creatinine (first 48 h)	0.96 mg/dl (0.66–1.29)	1.17 mg/dl (1.02–2.06)
	Urine output (24–48 h of life)	2.079 ml/kg/h (0.720–10.070)	1.72 ml/kg/h (0–4.24)

^a^ Damage of the brain on MRI scan 0 = no damge 1 = damage.

### 3.2 Predictive model development

Using a forward stepwise analysis, variables were incrementally introduced that were hypothesized to exert a positive influence on the outcome. We began with a single predictor - systemic injury - in **model 1**, which explained a moderate proportion of the outcome variance (R^2^ = 0.296, *p* = 0.001). Next, we added a second variable, **neurologic damage**, yielding **model 2**, which displayed improved explanatory power (R^2^ = 0.512, *p* < 0.001). In **model 3**, we incorporated **liver damage**, further enhancing the model fit (R^2^ = 0.548, *p* < 0.001). Finally, a fourth predictor, **renal damage** was included, resulting in **model 4**, which achieved the strongest performance (R^2^ = 0.621, *p* < 0.001). At each step, the inclusion of an additional predictor resulted in a discernible improvement in model fit, as evidenced by increasing explanatory power and statistical significance. This systematic procedure ultimately yielded a final model that captured the cumulative benefits of all relevant variables, thereby enhancing both its predictive accuracy and its clinical utility. Incorporating all four indices - liver damage, neurologic damage, systemic injury, and renal damage - the overall model demonstrated high statistical significance (ANOVA *p* = <0.001) and accounted for 62.1% of the variance in the outcome (R^2^ = 0.621). Among the individual predictors, the liver index (*β* = −0.726, *p* = 0.033), neurologic index (*β* = −0.401, *p* = 0.006), and systemic injury index (*β* = −1.042, *p* = 0.001) each showed a significant association with the outcome variable. Notably, the renal function index (*β* = 0.222, *p* = 0.098) exhibited a positive but non-significant correlation. Although the renal function index did not meet the conventional threshold for statistical significance, it was included in the final model based on its clinical relevance.

### 3.3 EPO-score - a scoring system for an early prediction of outcome

#### 3.3.1 EPO-score construction and parameter selection

Based on our analysis, we selected these 8 parameters, reflecting four different organ systems, to form a scoring system to predict outcomes (Table 5). Each parameter was graded across four levels of severity and all variables were scored on a symmetric scale ranging from 0 to 3 points. The EPO-Score (Early prognostic Outcome Score) for infants with perinatal asphyxia undergoing TH was developed by assigning points to abnormalities in the following postnatal laboratory values and examination results:

1) first arterial lactate after birth,
2) peak LDH within the first 48 h of life,
3) brain damage on MRI,
4) stage of HIE (classified according to Sarnat and Sarnat),
5) peak AST within the first 48 h of life,
6) peak ALT within the first 48 h of life,
7) peak serum creatinine within the first 48 h of life,
8) urine output between 24 and 48 h of life.

**Table 5:** EPO-Score.

Parameters	Values with units	Points
First arterial lactate	<3 mmol/L	0
	3.0–8.0 mmol/L	1
	8.01–13 mmol/L	2
	>13 mmol/L	3
Peak LDH within the first 48 h of life	<765 U/L	0
	765–1,000 U/L	1
	1,001–1,500 U/L	2
	>1,500 U/L	3
Brain damage on MRI	Normal MRI brain scan	0
	Brain damage on MRI brain scan	3
Stage of HIE (classified according to Sarnat and Sarnat)	Stage 0	0
	Stage 1	1
	Stage 2	2
	Stage 3	3
Peak AST within the first 48 h of life	<100 U/L	0
	100–200 U/L	1
	201–300 U/L	2
	>300 U/L	3
Peak ALT within the first 48 h of life	<52 U/L	0
	52–70 U/L	1
	71–90 U/L	2
	>90 U/L	3
Peak serum creatinine within the first 48 h of life	<0.8 mg/dl	0
	0.8–1.0 mg/dl	1
	1.01–1.2 mg/dl	2
	>1.2 mg/dl	3
Urine output 24–48 h after birth	>3 ml	0
	1.51–3 ml	1
	0.5–1.5 ml	2
	<0.5 ml	3

The included parameters were selected based on their relevance to clinical practice and previous research findings. The maximum score is 24 points, while the minimum is 0 points. The obtained total score using this EPO-Score was then compared to outcomes at one year of age, assessed by using the GMDS, the morbidity and the mortality data. For the description of outcome, the GMDS score obtained during the follow-up examination was used. Children who developed cerebral palsy or died during the follow-up period could not participate in the GMDS assessment. Due to the severity of their condition, we assigned a GMDS score of 0 to allow inclusion in the analysis.

#### 3.3.2 Comparison of EPO-scores between favorable and adverse outcome groups

**Figure 2:**
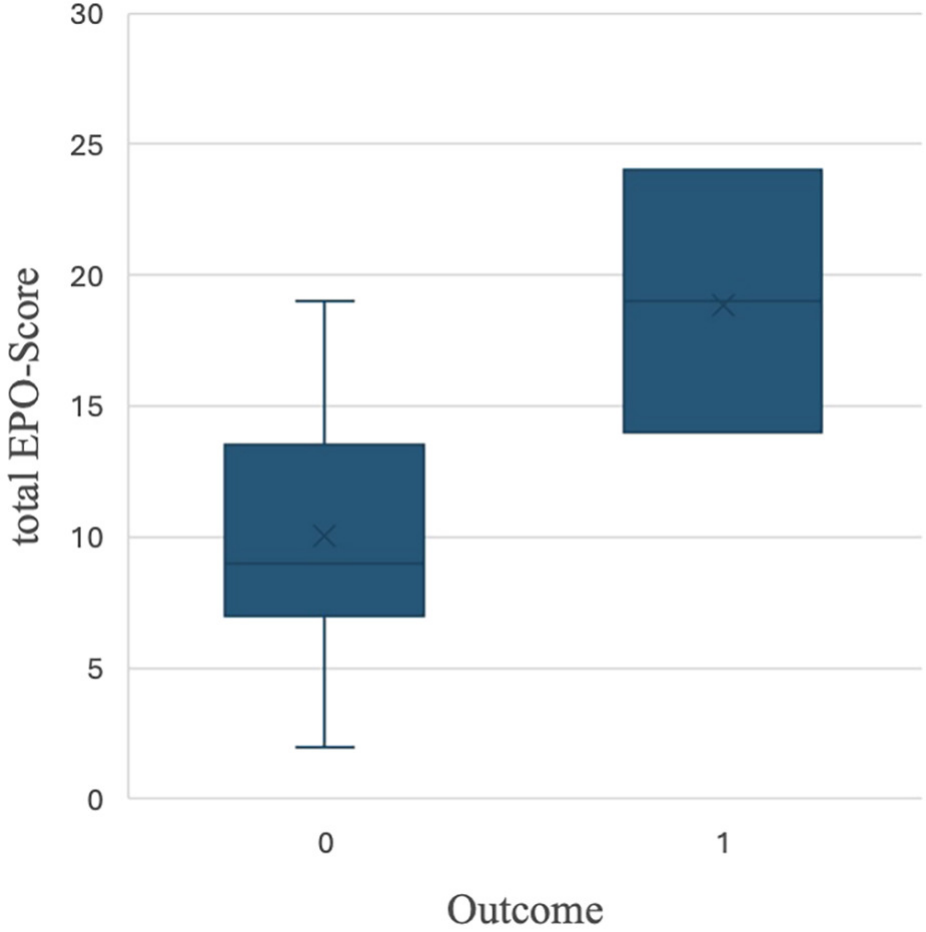
Boxplot of total EPO-score by outcome. 0 = favorable outcome; 1 = severe adverse outcome.

An overall EPO-Score was retrospectively calculated for evaluation purposes, an average score of 11 (2–24) was achieved among all infants. The mean EPO-Score was 9 (2–19) for the favorable outcome group vs. 19 (14–24) for the severe adverse outcome group (Figure 2). A significant correlation was found between the EPO-Score and the outcome at one year of age. The total EPO-Score as the sole predictor accounts for approximately 42% of the variance in the Outcome (R^2^ = 0.421). The model fit was highly significant (*p* < 0.001). Furthermore, we detected that a 1-point increase in the EPO-Score corresponded to a 5-point reduction in GMDS scores (B ≈ −5, *p* < 0.001).

## 4 Discussion

### 4.1 Development and predictive performance of the EPO-score

Perinatal asphyxia is a widespread clinical challenge and puts a strain on the entire body and all organ systems (9, 10, 16). The aim of this study was to identify parameters with predictive value reflecting different organ systems. For the clinical predictors, we combined biochemical parameters with the HIE-Score by Sarnat and Sarnat and brain imaging. Aiming to create a score that is easy to use, easy to interpret and cost-effective we created the EPO-Score that can be performed repeatedly. By combining various parameters into indices and incorporating these indices into a statistical model, we were able to demonstrate that this approach significantly improved the prediction of severe adverse outcome defined as death, cerebral palsy or a DQ < 78 on the GMDS. Based on this, we identified the parameters that were incorporated into the EPO-Score. The EPO-Score showed a strong correlation with neurodevelopmental outcomes at one year of age, measured using the Griffiths Mental Development Scales (GMDS) (15). Receiver Operating Characteristic (ROC) analysis confirmed its discriminatory power between infants with a favorable and severe adverse outcome with an area under the curve (AUC) of 0.926 (95% CI: 0.831–1.00), a sensitivity of 100%, and a specificity of 76%. The original statistical cut-off was calculated at 13.5 points. The practical threshold was adjusted to 14 points. In our cohort, all infants with a score of 14 or higher were identified as high-risk for severe adverse outcome. While previous studies have often focused on single biomarkers, the EPO-Score integrates multiple clinically relevant parameters into a composite tool that reflects the multi-systemic nature of perinatal asphyxia and offers meaningful support in early risk stratification.

### 4.2 Prognostic biomarkers and the role of multi-organ involvement in perinatal asphyxia

Elevated serum lactate levels in term infants are evidence of severe acidosis, which is part of the pathogenesis of perinatal asphyxia (17). Therefore, we included the first measured arterial lactate as being considered as an important biomarker for anaerobic glycolysis (18). Several authors have shown that infants with moderate or severe HIE have had significantly higher serum lactate levels than healthy infants or infants with mild HIE (19, 20). As a single biomarker, it was previously shown that high arterial lactate levels have a high negative predictive value regarding the neurological outcome (21). In addition to lactate, similar observations were made for LDH, as elevated LDH levels were significantly associated with poor neurological outcomes in infants with HIE (22).

While elevated lactate and LDH levels provide essential information about the extent of tissue injury, brain imaging offers crucial insights into potential cerebral damage. Given that the brain is highly vulnerable to hypoxia, brain imaging is an essential and proven method for early detection of possible brain damage (23, 24). Moreover, MRI has been used before and during the hypothermia era to assess the severity of damage. Various authors do not only describe the clearly positive effect of therapeutic hypothermia on brain damage (5, 25), but also emphasize its prognostic value in describing the probability for disability or death (6, 8, 26). This underscores why MRI brain scans are a critical component of our scoring and the results present the correlation between detected brain damage and a poorer outcome. Besides imaging, clinical classification plays a key role in assessing the severity of HIE. The grading system by Sarnat and Sarnat, introduced in 1976, is widely used to categorize infants based on the severity of HIE (12). It guides treatment decisions, but previous studies have also demonstrated its predictive value for neurological development and long-term outcome (27–29). The severity of HIE, as reflected in this grading system, significantly correlates with prognosis.

One of the organs most commonly affected by perinatal asphyxia is the liver. Liver damage is seen in approximately 42%–48% of newborns with perinatal asphyxia. Liver enzymes such as ALT and AST play an important role in assessing the extent and severity of hypoxic ischemic encephalopathy and liver damage (30, 31). Recent studies suggest that high levels of AST and ALT in the first 24–72 h of life correlate with severe HIE and worse long-term outcomes, which supports our findings (32). In addition to liver involvement, the kidneys are significantly affected in cases of perinatal asphyxia. Epidemiological data show that 50%–70% of asphyxiated newborns suffer from an impaired renal function and an acute kidney injury (AKI) (33, 34). AKI is often characterized by reduced urine output and high serum creatinine levels (35). Several studies have reported an association between serum creatinine levels and neurodevelopmental outcomes. Alaro et al. discovered that the prevalence for AKI was 15 times higher in infants with HIE III than HIE I and mortality was even 24 times higher when infants were diagnosed with AKI (36). Moreover, oliguric renal failure is associated with a significantly higher mortality rate than non-oliguric renal failure (33).

In the past, numerous studies focused on individual prognostic parameters, each related to a single organ system. More recently, efforts have shifted towards the aspect of multi-organ involvement in patients with perinatal asphyxia (16), which contributes to outcome prediction. Making predictions based on a single parameter carries the risk of a limited perspective and can lead to inaccurate predictions. Relying on a single factor ignores the complexity of the situation and may overlook other important variables influencing the outcome, potentially resulting in biased or unreliable predictions.

In studies addressing multi-organ involvement, particular attention has been given to the cardiovascular, neurological, renal, hepatic, and hematological systems. These organ systems have demonstrated some degree of significance in predicting outcomes. Dysfunction of these systems are often associated with worse outcomes. However, despite these findings, many studies have not linked these parameters together in a comprehensive, integrated model and therefore ultimately limiting their clinical applicability (37). Scoring systems in medicine have been proven valuable for assessing a patient's clinical status by clarifying individual performance and by initially assessing the urgency of a required treatment. Few authors have devised scoring systems attempting to encompass numerous organ systems and incorporate various laboratory parameters, thus enhancing detail but also complexity and usability challenges (9, 10). Razif et al. conducted a systematic review of multi-organ scoring systems in neonatal encephalopathy, including twelve studies published between 1989 and 2022. Although the studies showed substantial heterogeneity—varying enrolment periods, organ definitions, cut-off values and follow-up intervals—every paper confirmed that greater multi-organ dysfunction correlated with higher mortality and poorer neurodevelopmental outcomes. The scores by Alsina et al. and Sweetman et al. were highlighted as the most promising because they defined clear cut-off thresholds (37). Alsina et al. incorporated six organ systems and 23 clinical and laboratory parameters to establish a significant correlation between multi-organ involvement and stages of HIE. Unfortunately, the outcome was not considered, as their follow up period ends with three days of age (10). More recently, Sweetman et al. developed the multi-organ dysfunction scoring in neonatal encephalopathy (MODE-Score) consisting of 17 parameters. They identified a correlation between their total score and HIE stage. Additionally, they compared their findings with outcomes at follow-up after 24 months and observed a high predictive value for their scoring system (9). Nevertheless, both studies used lots of variables for their score, which makes them more detailed, but also more complicated to use in the daily clinical practice. In 2024, Estiphan et al. developed domain-specific risk scores for Bayley-III outcomes that relied on just five routinely available variables: 5-minute APGAR- score, initial HIE stage, neurological status at discharge (days 5–7), EEG-verified seizure burden, and MRI lesion pattern. In their cohort, the presence of ≥3 abnormal factors predicted cognitive deficits (PPV 60%, NPV 95%) and language deficits (PPV 85%, NPV 90%) at 24 months, whereas ≥2 factors predicted motor impairment with a PPV of 71% and an NPV of 100%. Because the score draws exclusively on clinical examination and imaging, it delivers a rapid, neuro-focused risk estimate; however, the omission of laboratory markers means that multi-organ involvement is not captured (38).

### 4.3 Clinical implications, study limitations and future directions

The early identification of infants at risk to have a severe outcome plays a significant role in terms of clinical decisions, subsequent follow-up care and support for both newborns and their families. Supporting and preparing parents during this difficult time is an important aspect of doctor-patient communication and should be an important part of everyday clinical practice. With the EPO-Score we present a scoring system that is easy to use and may help to categorize infants. It helps to identify infants in need for specialized follow-up care and therefore ensures improved care opportunities. Consisting of only seven common parameters and image display, it is practical, low-cost and should be useful for a wide range of institutions. However, the EPO-Score as introduced by our study requires further validations as there are a few limitations: (1) As we conducted this study retrospectively, the data collection was not initialized especially for this study, which is why we had to face incomplete and missing neonatal data and some patients were lost in the follow-up. (2) We chose to assess the neurocognitive outcome at 12 months of age. This should be re-evaluated at later date e.g., two years of age or at school age. (3) The present cohort is small and from a single center. As a result, only apparent performance could be reported, and some over-optimism was inevitable. Robust bootstrap resampling and external validation in larger, more diverse populations are therefore essential before the EPO-Score can be considered for routine clinical use. (4) Only seven severe-adverse events were observed for eight predictors (<1 event per predictor), increasing the likelihood of residual over-fitting. (5) EEG monitoring was performed in all cases before and during TH treatment, but the archived traces were not accessible for quantitative review, therefore seizure burden and background patterns, both known prognostic markers (39, 40), could not be tested as candidate predictors in the present model. (6) Ethnicity and socio-economic status were not recorded, preventing a formal fairness assessment across demographic groups; future multicenter studies in more diverse populations are therefore essential before clinical implementation.

Looking back and moving forward, perinatal asphyxia remains a major challenge in terms of outcome prediction, strategies, and long-term follow-up care. Although neonatal medicine has made substantial progress, accurate early risk stratification remains difficult. Reliable, user-friendly tools are urgently needed to support clinical decision-making and to optimize individualized care strategies. The EPO-Score offers a promising approach by combining clinically relevant and readily available parameters into a simple system that facilitates early identification of high-risk infants. We plan to apply the EPO-Score prospectively to new cohorts of asphyxiated infants and correlate the results with detailed long-term follow-up data. Furthermore, we would welcome the opportunity for the EPO-Score to be tested in multiple Neonatal Care Units, thereby validating it across diverse clinical settings and larger populations. Broader application would not only strengthen the scientific evidence supporting its predictive value but could also contribute to the development of standardized follow-up protocols for affected infants. Ultimately, we hope that the EPO-Score will become a valuable component in the ongoing efforts to improve the care and prognosis of infants suffering from perinatal asphyxia.

## Data Availability

The raw data supporting the conclusions of this article will be made available by the authors, without undue reservation.
