# Supplementary material for: Development of the EPO-Score – a multivariable tool to predict adverse outcome in infants with perinatal asphyxia undergoing therapeutic hypothermia – a retrospective study
Source: Front Pediatr. 2025 Aug 6;13:1627300. doi: 10.3389/fped.2025.1627300 (PMC12364892; doi:10.3389/fped.2025.1627300)
Supplement: Supplementary file 1 [file Table1.pdf]

S1: Overview of the criteria for therapeutic hypothermia (12-14)

Inclusion criteria for hypothermia treatment:

- Gestational age ≥36+0 weeks
- Start of treatment within 6 hours after birth
- No severe intracranial haemorrhages or severe congenital malformations
- Diagnosis of perinatal asphyxia **and** at least one neurological criterion must be fulfilled

**Asphyxia criteria**	**Neurological criteria**
• Fetal distress (pathological CTG **or** fetal bradycardia **or** fetal blood gas analysis with arterial lactate > 4,7mmol/l **or** green amniotic fluid) **and**<br>• pH ≤ 7.0 **or** Base Excess ≥ - 16mmol/l **or**<br>• 5-minute APGAR-Score < 6	• Pathological aEEG (EEG) **or**<br>• Clinical signs of moderate or severe HIE in at least three of the six clinical categories by Sarnat and Sarnat

Classification of hypoxic-ischemic encephalopathy (HIE) after perinatal asphyxia, modified from Sarnat & Sarnat and Shankaran (12,14)

	Stage 1 (mild)	Stage 2 (moderate)	Stage 3 (severe)
1)Level of consciousness	Awake	Lethargic	Stupor / coma
2) Activity	Normal	Decreased	None
3) Muscle stretch reflexes / Posture	Exaggerated, posture normal	Exaggerated, distal flexion or full extension	Diminished / absent; flaccid posture
4) Muscle tone	Normal	Mild hypotonia	Flaccid
5) Primitive reflexes<br><br>Suck<br>Moro	 <br>Active<br>Exaggerated	 <br>Weak<br>Incomplete	 <br>Absent<br>Absent

Grasp<br>Vestibulo-ocular reflex	Normal/exaggerated<br>Normal	Exaggerated<br>Exaggerated	Absent<br>Reduced/absent
6) Autonomic function<br><br>Pupils<br>Heart rate<br>Respiration	 <br>Dilated/reactive<br>Normal/ tachycardia<br>Regular	 <br>Constricted, reactive<br>Bradycardia<br>Periodic, irregular	 <br>Irregular, wide, non-reactive<br>Bradycardia, variable<br>Apnoea
Seizures	None	Common	Common
EEG	Normal	Amplitude depression (< 25 µV), periodic or paroxysmal activity	Periodic or isoelectric trace
aEEG	Normal<br>upper limit: > 10 µV<br>lower limit: > 5 µV	Moderately abnormal<br>upper limit: > 10 µV<br>lower limit: < 5 µV	Severely abnormal<br>upper limit: < 10 µV lower limit: < 5 µV<br>± burst suppression

S1: Supplementary File S1: Overview of the criteria for therapeutic hypothermia
