# Supplementary material for: Development of the EPO-Score – a multivariable tool to predict adverse outcome in infants with perinatal asphyxia undergoing therapeutic hypothermia – a retrospective study
Source: Front Pediatr. 2025 Aug 6;13:1627300. doi: 10.3389/fped.2025.1627300 (PMC12364892; doi:10.3389/fped.2025.1627300)
Supplement: Supplementary file 2 [file Table2.pdf]

Time in hours	Event	Blood gas analysis	Blood[1]	Urine output[2]	HIE stage	EEG	Clinical examination	Imaging
0	Start of cooling	X	X	X	X	X	X	Cranial ultrasound, doppler sonography, echocardiography
3		X		X		X	X	
6		X	X	X		X	X	
12		X	X	X		X	X	
18			X	X		X		
20		X		X		X	X	
22				X		X		
24			X	X		X		Cranial ultrasound, doppler sonography, echocardiography
26				X		X		
28		X		X		X	X	
30				X		X		
32			X	X		X		
34				X		X		
36		X		X		X	X	
38				X		X		
40			X	X		X		
42				X		X		
44		X		X		X	X	
46				X		X		
48			X	X		X		Cranial ultrasound, doppler sonography
50				X		X		
52		X		X		X	X	
54				X		X		
56			X	X		X		

58				X		X		
60		X		X		X	X	
62				X		X		
64			X	X		X		
66				X		X		
68		X		X		X	X	
70				X		X		
72	End of cooling		X	X				Cranial ultrasound, doppler sonography
Day 4		X		X			X	
Day 5		X		X			X	(MRI)[3]
Day 6		X					X	(MRI)[3]
Day 7		X					X	MRI
Discharge							X	

[1] Blood: lactate, pH, BE, CK, LDH, AST, ALT, creatinine, urea and Quick
[2] Urine output was documented continuously for 5 days
[3] MRI was performed at day 5-7 after completed cooling

S2: Supplementary Table S2: timeline of measurements and clinical assessments
