# Supplementary material for: Development of the EPO-Score – a multivariable tool to predict adverse outcome in infants with perinatal asphyxia undergoing therapeutic hypothermia – a retrospective study
Source: Front Pediatr. 2025 Aug 6;13:1627300. doi: 10.3389/fped.2025.1627300 (PMC12364892; doi:10.3389/fped.2025.1627300)
Supplement: Supplementary file 3 [file Table3.pdf]

	Variable	Unit	% Missing	Retained	Reason for exclusion
1	Sarnat and Sarnat Score	/	0%	yes	
2	pH[ab]	/	0%	yes	
3	BE[ab]	mmol/l	0%	yes	
4	APGAR [a]<br>1min, 5min, 10min	/	15,6%, 18,75%, 21,8%	yes	
5	First arterial lactate[a]	mmol/l	0%	yes	
6	Lactate 6h[a]	mmol/l	0%	yes	
7	Lactate 12h[a]	mmol/l	0%	yes	
8	Lactate 18h[a]	mmol/l	0%	yes	
9	Lactate 24h[a]	mmol/l	0%	yes	
10	Lactate 30h[a]	mmol/l	0%	yes	
11	Lactate 36h[a]	mmol/l	0%	yes	
12	CK [ac]	U/l	0%	yes	
13	LDH[ac]	U/l	0%	yes	
14	AST[ac]	U/l	0%	yes	
15	ALT[ac]	U/l	0%	yes	
16	Serum creatinine[ac]	mg/dl	0%	yes	
17	Urea[ac]	mg/dl	0%	yes	
18	Quick[d] at admission	%	0%	yes	
19	Urine output[a]<br>0-24h<br>24-48h<br>48-72h	ml/kg/h	0%	yes	
20	Duration of mechanical ventilation[a]	h	0%	yes	
21	Duration of catecholamines[a]	h	0%	yes	

22	MRI brain damage	/	0%	yes	
23	Troponin I [ac]	ng/l	62,5%	no	>25% missing
24	CK-MB[ac]	U/l	68,75%	no	>25% missing
25	BNP[ac]	ng/l	81,25%	no	>25% missing
26	S100 Protein[a]	µg/l	90,625%	no	>25% missing
27	Ferritin[e]	µg/l	62,5%	no	>25% missing

[a] Data expressed as [a] median (IQR), [b] Data collected within the first 3h of life, [c] Data collected within the first 48 hours of life
[d] Data expressed in %, [e] Data collected for 10 days

S3: Supplementary Table S3 Full list of candidate predictors
